# Surfactant-Assisted Assembly of Dipeptide Forming a Broom-like Structure

**DOI:** 10.3390/molecules27154876

**Published:** 2022-07-29

**Authors:** Yunping Wei, Jie Zhang, Xingcen Liu

**Affiliations:** Key Laboratory of Colloid and Interface Chemistry of the Ministry of Education, School of Chemistry and Chemical Engineering, Shandong University, Jinan 250100, China; ypwei@sdu.edu.cn (Y.W.); jiezhang_sdu2015@163.com (J.Z.)

**Keywords:** dipeptide, broom-like structures, assembly, surfactant

## Abstract

Understanding the influence of surfactants on the assembly of peptides has a considerable practical motivation. In this paper, we systematically study the anionic surfactant-assisted assembly of diphenylalanine (FF). FF forms broom-like structures in a concentration of sodium cholate (NaC) around the CMC, and assembles into linear and unidirectional rods in the presence of low and high surfactant concentrations. FF’s improved hydrogen bonding and controlled assembly rates are appropriate for other anionic surfactants. At this stage, the use of FF as the simplest protein consequence can be helpful in the investigation of further protein–surfactant interactions.

## 1. Introduction

Natural and spontaneous self-organized amyloid fibril assemblies have encouraged the study of peptides as bio-supramolecular structural building blocks [1,2,3]. The behavior of peptides, which can self-assemble into various nanostructures, has attracted much attention because of their applications in biomedical, bioengineering and drug production [4,5,6,7,8]. In contrast to other supramolecular structural motifs, the unique and desirable features of peptides include their chemical diversity, sequence-specific secondary structures, biomolecular recognition, high biocompatibility, and ease of synthesis [9,10,11,12,13,14,15]. As a minimal-recognition module, the dipeptide Phe-Phe (FF), which can easily assemble into various ordered structures, is the most studied because of its mechanical, electrical and optical properties [16,17,18,19,20,21]. Assembled structures and functionalities based on FF are usually related to the cooperation or compromise of multiple inter- and intramolecular interactions, such as hydrogen bonds, hydrophobic effects, π-π interactions, and electrostatic forces [22,23,24,25].

Multiple strategies have been successfully used for the assembly of peptides, such as solvent-induced, sonication-assisted, ionic, vaporization-induced, coordination-assisted, and surfactant-assisted assembly, as well as organic or water gelation, etc. [26,27,28,29,30,31,32]. Surfactants change the solvent environment and modulate interactions with peptides due to their hydrophilic head group and hydrophobic tail groups. In addition to hydrogen bonds, the main interactions between surfactants and peptides are hydrophobic and electrostatic interactions. The supramolecular association of peptides and surfactants leads to a broader range of structures, including precipitates, gels, micelle-like aggregates, complexes, and coacervates [33,34,35,36,37,38].

Although previous reports focused on the organization of the various FF structures, the influence of surfactants on FF-assembly remains unknown, and exerting control over FF-assembly is still very important. Therefore, in this study, we analyzed the interactions between FF and surfactants by studying the microstructures of FF assembly in the presence of different surfactants. We started by examining an anionic surfactant sodium cholate (NaC). Then, we studied other anionic and cationic surfactants. The appropriate concentrations of anionic surfactants assisted FF’s assembly into a broom-like structure. We also proposed a hypothesis to explain the interactions between surfactants and peptides.

## 2. Results and Discussion

### 2.1. Assembly Induced by NaC (Phase Behavior)

**Figure 1:**
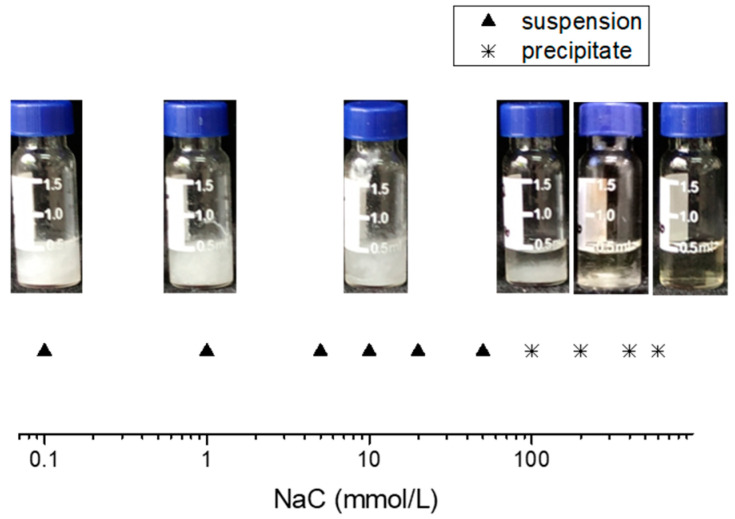
The phase diagram of FF/NaC mixtures with a concentration of FF of 10 mmol/L.

FF can self-assemble into hexagonal crystalline microtubes and microrods in water [39]. Here, we investigated the aggregation behaviors of FF/NaC mixtures in order to explore the effect of NaC on FF assembly. Figure S1 shows the chemical structure of NaC and FF. Figure 1 shows the phase diagram of the increasing concentrations of NaC solution added to the freshly prepared FF/HFIP solution. The mixtures appear as a suspension at a low concentration of NaC. When the concentration is up to 100 mmol/L, phase separation occurs and the clear solution and precipitates appear at the bottom. In addition, the aggregation speed decreases as the concentration of NaC increases, as shown in Figure S2. After 30 min, all of these mixtures achieve equilibrium. When the concentration is below 1 mmol/L, the effects on the speed are negligible. The aggregation growth slows down obviously when the concentration rises to 5 mmol/L. In particular, at concentrations over 500 mmol/L, the clear solution and tiny precipitates reach a balance after one month.

**Figure 2:**
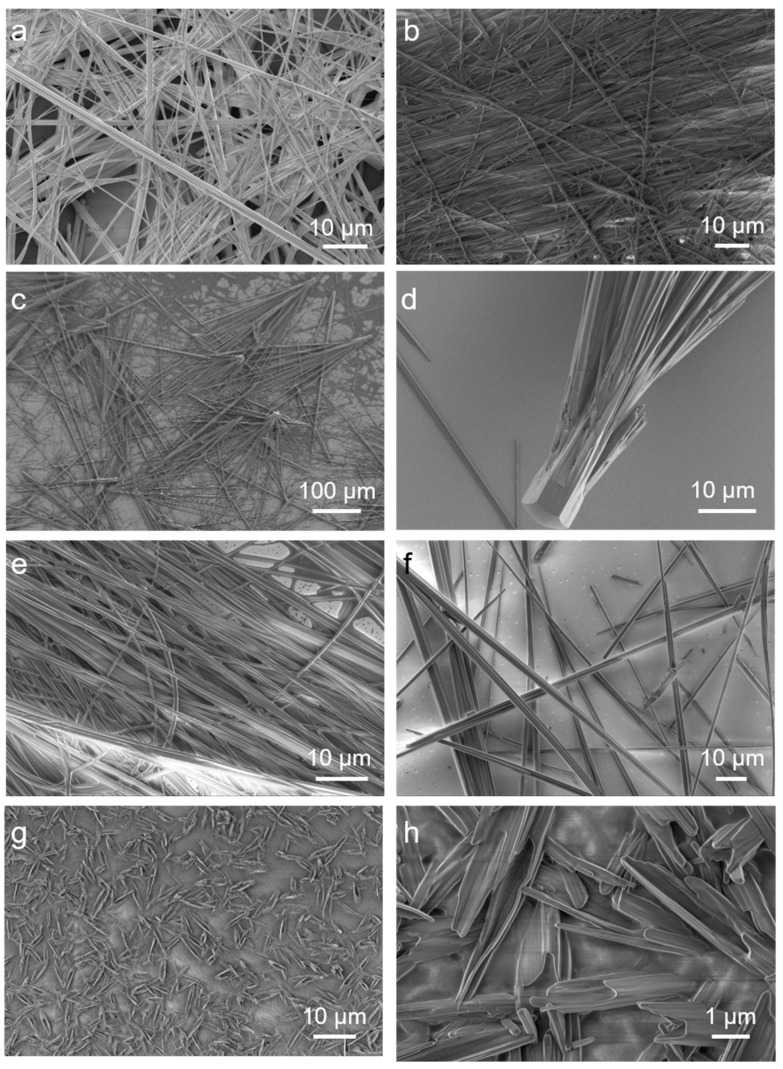
The SEM images of FF/NaC mixtures at NaC concentrations of (**a**) 0, (**b**) 1, (**c**,**d**) 5, (**e**) 20, (**f**) 300, and (**g**,**h**) 500 mmol/L, with the fixed FF concentration of 10 mmol/L.

We performed optical microscopy observations in an aqueous solution to show the morphologies of the assembled aggregates above the fixed FF concentration of 10 mmol/L (Figure S3). The microstructures of naturally dried samples were also investigated by SEM and TEM observations in Figure 2 and Figure S4, which are in agreement with the optical microscopy results. Small amounts of NaC barely affect the aggregates, which remain the same ultralong microrods that grow up to several millimeters in length linearly and unidirectionally in pure water (Figure 2a,b). However, as the concentration increased to approximately 5 mmol/L, we observed broom-like structures (Figure 2c) with lengths from 400 to 1000 μm and a stability of up to one month. The video in the Supporting Information shows the growth process of the broom-like structures. It proves that the assembly grows from the nucleus and then starts to become branched. After that, the branches gradually grow longer, and they produce the final structure in less than two minutes. Furthermore, the ends of the broom-like crystals in Figure 2d clearly show the same hexagonal shapes as the FF microrods in water. Broom-like rods appear when adding FF into different concentrations of NaC solutions from 5 to 12 mmol/L (Figure S5). After the NaC concentration rises to 20 mmol/L, it still shows ultralong rods (Figure 2e,f). With an increasing NaC concentration, the amount of FF aggregations decreases remarkably, as the images in Figure 1 show. At an NaC solution concentration of 500 mmol/L, the FF failed to assemble into rods, and after one month the fragments left without forming any rods (Figure 2g,h). These results confirm that the concentration of NaC has a strong influence on the assembly of FF.

**Figure 3:**
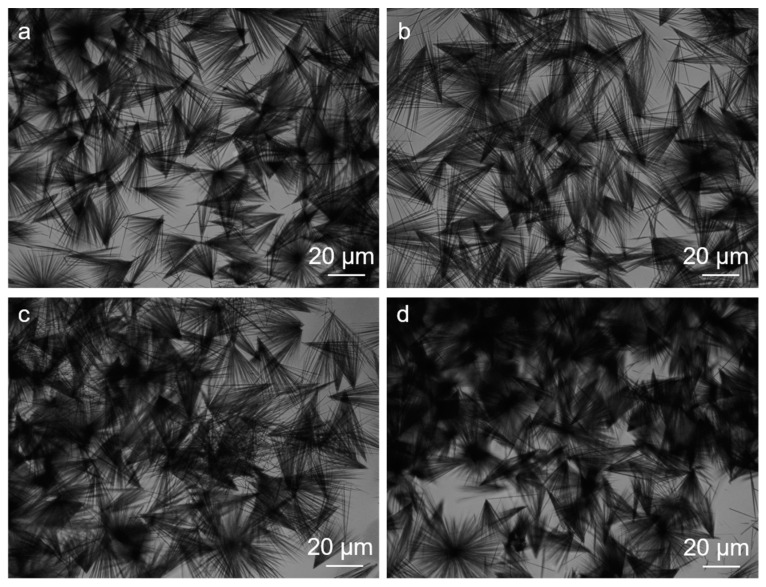
The images of the FF/NaC mixtures at a NaC concentration of 8 mmol/L with FF concentrations of (**a**) 30, (**b**) 40, (**c**) 50, and (**d**) 60 mmol/L, as given by optical microscopy.

Then, we increased the concentration of FF with a fixed NaC at 10 mmol/L. When the FF is above 30 mmol/L, the aggregates assemble smaller 20–50 μm-long broom-like crystals (Figure 3). The broom-like structures and sizes do not change even when we increase the FF concentration to the maximum value (about 60 mmol/L).

### 2.2. The Secondary Structures of the FF/NaC Mixture

**Figure 4:**
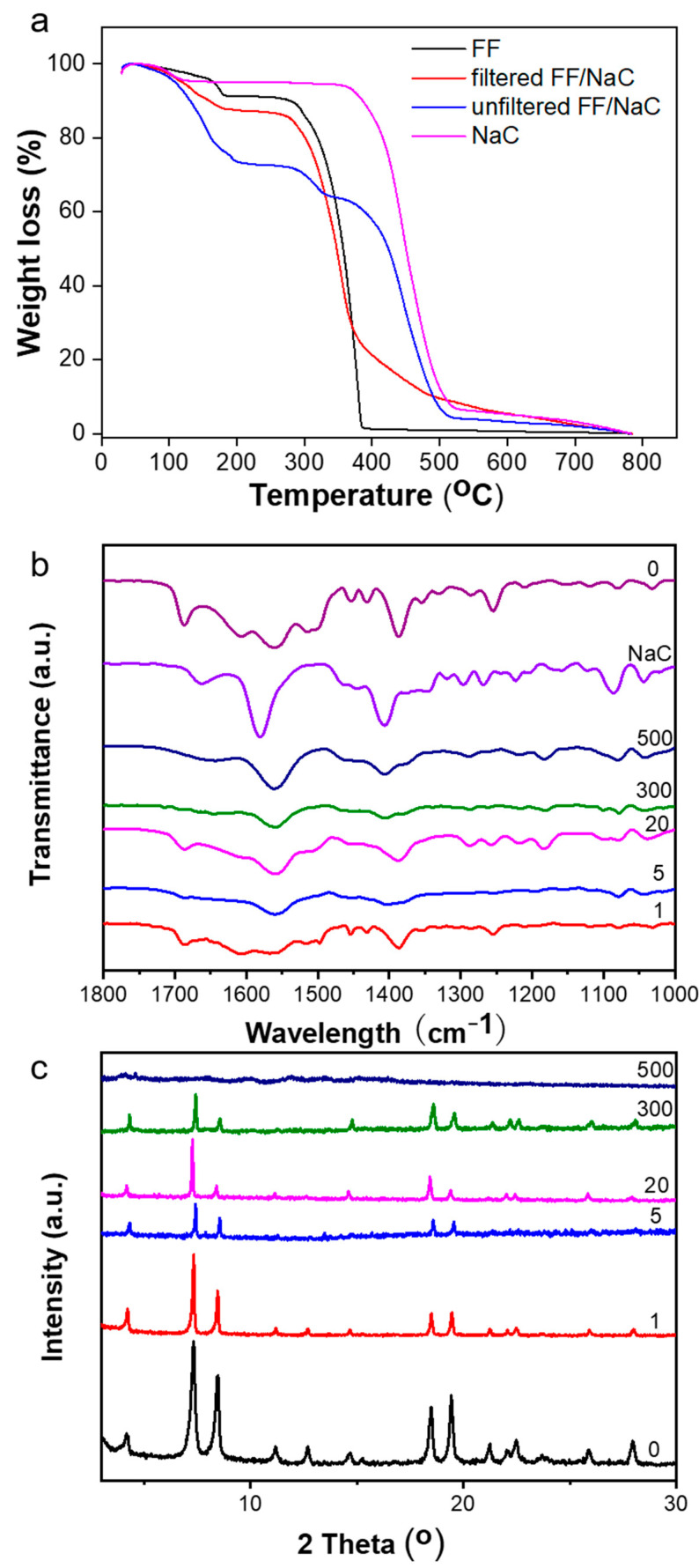
(**a**) The TGA curves of pure FF, pure NaC, and FF/NaC mixtures. (**b**) FTIR and (**c**) XRD spectra of FF/NaC mixtures at NaC concentrations of 0, 1, 5, 20, 300 and 500 mmol/L, with a fixed FF concentration of 10 mmol/L.

In addition, we probed the thermal stability by thermogravimetric analysis (TGA). We present the thermal degradation curves of pure FF, pure NaC, and mixtures before and after filtration in Figure 4a. The mixtures before filtration are comprised of FF/NaC, and the FF and NaC monomers dissolve in water. After filtration, the mixtures are only composites of FF/NaC. The two-step degradation of the filtered FF/NaC mixture shares the same temperatures (around 180 °C) with pure FF. For the filtered FF/NaC mixture, we only measured the broom-like structures after drying, and we observed a slight amount of NaC degradation with 20% weight left, indicating that NaC plays a role inside the filtered FF/NaC mixtures. Before filtration, the FF/NaC mixture shows a remarkable amount of NaC degradation, with a weight loss of 60%, which proves that more NaC monomers are present in the solution instead of taking part in FF rods. This result proves that a small amount of NaC takes part in the assembly of the FF/NaC mixture, in addition to the larger amount of NaC dispersed in the solution.

We performed Fourier transform infrared spectroscopy (FTIR) and X-ray diffraction (XRD) to illustrate the effects of NaC on the secondary structures and molecular interactions of this system. The spectra are shown in Figure 4b,c. The FTIR results demonstrated amide I and II absorption bands near 1675 cm^−1^ and 1602 cm^−1^, respectively, confirming the β-sheet secondary structures of the FF rods. The XRD spectra of the FF rods with or without NaC share the same sharp peaks, indicating the same hexagonal structure of the FF microrods [39]. However, we observed no sharp peaks for the mixture with 500 mmol/L NaC, which is in agreement with the fragments of the FF aggregates. Therefore, the secondary structures of FF/NaC mixtures remain unchanged, while the assemblies form differently in the presence of NaC.

### 2.3. The General Observation of Different Surfactants

We analyzed more anionic surfactants such as sodium dodecyl sulphate, dodecyl sodium sulfonate, and sodium deoxycholate (Figure S6). It turns out that they all formed the broom-like structures near their CMC. However, only long rods left upon the change to cationic surfactants such as cetyl trimethyl ammonium bromide (CTAB), tetradecyl trimethyl ammonicum bromide (TTAB), and cetanecyl trimethyl ammonium chloride (CTAC). These results demonstrate that the interactions between anionic surfactants and FF play an important role in the formation of broom-like structures.

### 2.4. Mechanism of the Formation of the Assemblies

**Figure 5:**
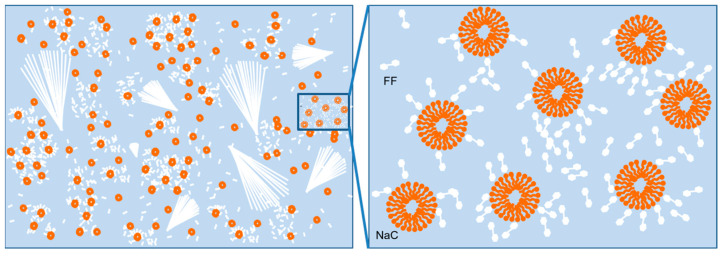
The dispersion, nucleation and growth of FF with an NaC concentration around the CMC.

The typical nucleation and growth scenario is appropriate for this system. Nucleation starts when the FF concentration exceeds supersaturation and the nucleation barrier is crossed. Next is the aggregation of nuclei and the growth process. With a pure FF/water system, the FF molecules aggregate near the nuclei and support the growth of long microrods. Hydrogen bonding is a key force, and water mediation is necessary in order for FF to grow microrods [39,40,41]. We proposed the following hypothesis regarding the presence of NaC: the synergistic effects of hydrogen-bonding interactions and the controlled FF growth rate (Figure 5). The carboxylate ions of NaC can form hydrogen bonding with the carboxy group of FF, and they have electrostatic repulsion from the carboxylate ions of FF, which the cationic surfactants do not have. In addition, when the NaC concentration is around its CMC, plenty of micelles form in the solution [42,43,44] and the hydrophobic residues of FF molecules lead to a narrow growth space for aggregates, which in turn increasing the local FF concentration. The increasing accumulation of FF improves the nucleation and growth rate, which is where the dendritic growth takes place in the formation of a broom-like assembly [45]. At low NaC concentrations, the NaC molecule disperses in the solution as an ionic state or monomer, which has little influence on FF assembly. When the NaC concentrations keep increasing, the stabilization effect dominates to decrease the amount of FF nuclei and accumulation, resulting in line structures, which is in agreement with the images in Figure 1.

## 3. Materials and Methods

### 3.1. Materials

FF and 1,1,1,3,3,3-hexafluoro-2-propanol (HFIP) were bought from Sigma-Aldrich. All the surfactants were sodium dodecyl sulphate, dodecyl sodium sulfonate, and sodium deoxycholate. The cetyl trimethyl ammonium bromide (CTAB), tetradecyl trimethyl ammonicum bromide (TTAB), and cetanecyl trimethyl ammonium chloride (CTAC) were bought from the Aladdin company, with a purity of 95–98%.

### 3.2. Preparation of the Samples

All of the samples were prepared as follows: a freshly prepared FF/HFIP solution (0.32 mol/L, 10 µL) was diluted to a final FF concentration of 10 mmol/L by oscillating after adding different surfactant solutions. Then, all of the samples were freeze-dried for the next characterizations.

### 3.3. Measurements and Characterizations

The optical microscope (Scope A1 from ZEISS, SLMPLN50× from Olympus, NA = 0.35) measurements were taken with the solution or the dried fibers on the glass slides.

Scanning electron microscope (SEM) measurements were taken on JEOL JSM6700F or Hitachi S-4800 field-emission scanning electron microscopes. The samples with the gold coating were transferred onto the microscope stage and examined at 10 kV.

The transmission electron microscopy (TEM) images were obtained on a JEOL JEM-1400 transmission electron microscope (120 kV). A drop of the dispersed solution of the samples was dropped onto a TEM grid (a copper grid with a 200 mesh) and then dried for observation. Images were recorded with a Gatan multiscan charge-coupled device (CCD) for the collection and processing of digital micrographs. 

Thermogravimetric analysis (TGA) measurements were performed at DSC 822e (Piscataway, NJ, USA) with a scanning speed of 10 °C·min^−1^ over 50–800 °C under a nitrogen atmosphere.

Fourier transform infrared (FTIR) spectra were carried out on a VERTEX-70/70v FT-IR spectrometer (Bruker Optics, Germany) using a KBr pellet method.

X-ray diffraction (XRD) measurements were completed on a DMAX-2500PC diffractometer with Cu Kα radiation (λ = 0.15418 nm) and a graphite monochromator. Samples were examined within 1–30° in the 2θ mode at a speed of 1° min^−1^.

## 4. Conclusions

In conclusion, we have studied the effects of surfactants on the assembly of FF. The anionic surfactants have a considerable influence on the forms taken by FF—from long, linear rods to broom-like structures—by modulating the surfactant concentration around its CMC. The broom-like structures became smaller and more unified when the FF concentration increased. These effects are mainly influenced by the hydrogen between the carboxylate ions of anionic surfactants and the carboxy groups of FF molecules. Moreover, narrow spaces affected the FF assembly rate due to plenty of micelles being formed. Our findings should be useful for understanding the interactions between surfactants and peptides, and for the provision of further insight into biological amphiphile–protein interactions.

## Data Availability

The data is contained within the article or Supplementary Materials.

## Supplementary Materials

The following supporting information can be downloaded at: https://www.mdpi.com/article/10.3390/molecules27154876/s1. Figure S1: The molecular structure of FF and NaC. Figure S2: The images of FF/NaC mixtures with time changes at NaC concentrations of 0, 1, 5, 20, 100, and 300 mmol/L (from left to right). Figure S3: The images of FF/NaC mixtures at NaC concentrations of 0, 1, 5 and 150 mmol/L. Figure S4: The TEM images of FF/NaC mixtures at NaC concentrations of (a) 0, (b) 1, (c) 5, (d) 20, (e) 300 and (f) 500 mmol/L. Figure S5: The images of FF/NaC mixtures at NaC concentrations of 5, 7, 10, and 12 mmol/L, where the c(FF) is 10 mmol/L. Figure S6: The images of different FF/surfactant mixtures, with the concentrations of the surfactants being near their CMC. Video: The growth of the broom-like structures by the NaC-assisted assembly of FF.

## Sample Availability

Samples of the compounds are available from the authors.
