# Supplementary material for: Surfactant-Assisted Assembly of Dipeptide Forming a Broom-like Structure
Source: Molecules. 2022 Jul 29;27(15):4876. doi: 10.3390/molecules27154876 (PMC9369827; doi:10.3390/molecules27154876)
Supplement: Supplementary file 1 [file molecules-27-04876-s001.zip › molecules-1814093-supplementary.pdf]

molecules

MDPI

Supplementary Materials

Surfactant-Assisted Assembly of Dipeptide Forming a Broom-Like Structure

Yunping Wei, Jie Zhang and Xingcen Liu *

Key Laboratory of Colloid and Interface Chemistry of the Ministry of Education, School of Chemistry and Chemical Engineering, Shandong University, Jinan 250100, China; ypwei@sdu.edu.cn (Y.W.); jiezhang_sdu2015@163.com (J.Z.)
* Correspondence: liuxingcen@sdu.edu.cn

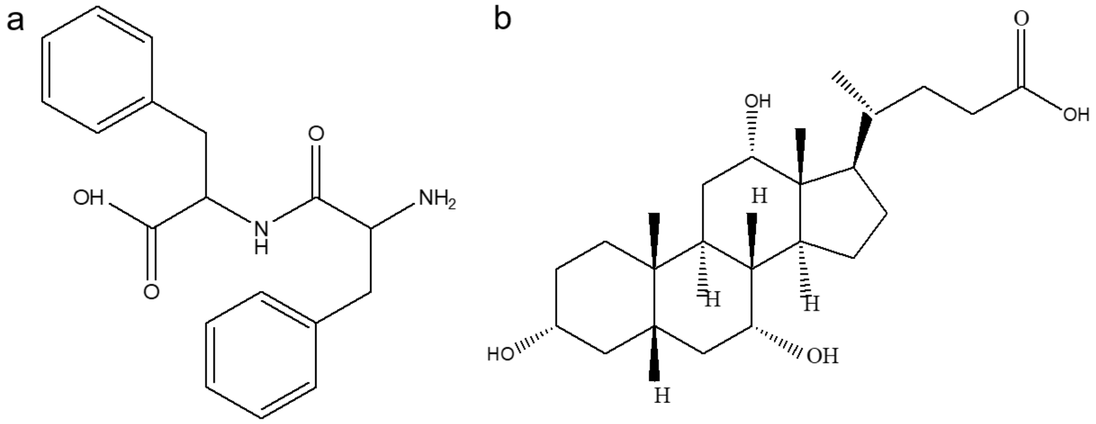

Figure S1. The molecular structure of (**a**) FF and (**b**) NaC.

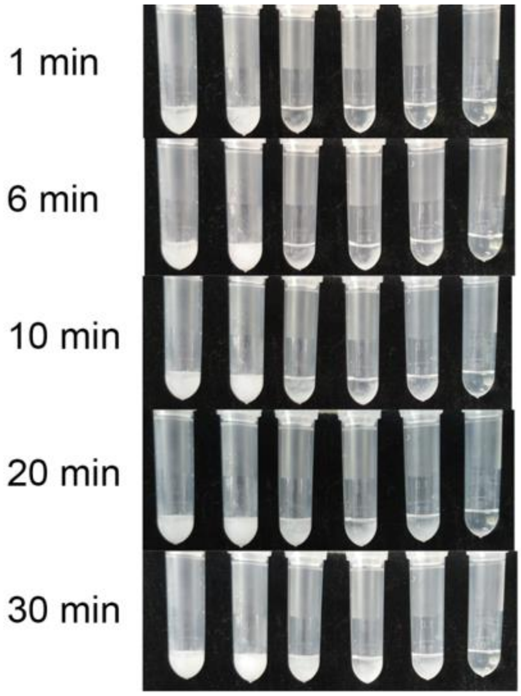

Figure S2. The images of FF/NaC mixtures with time changes at NaC concentration of 0, 1, 5, 20, 100, and 300 mmol/L (from left to right) and the fixed FF concentration of 10 mmol/L.

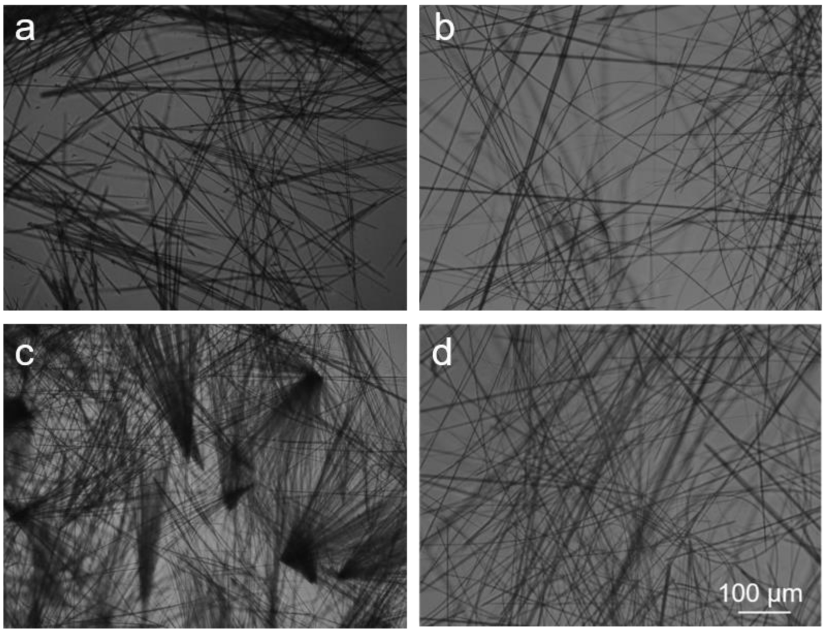

Figure S3. The images of FF/NaC mixtures at NaC concentration of (a) 0, (b) 1, (c) 5 and (d) 150 mmol/L with the fixed FF concentration of 10 mmol/L by the optical microscopy.

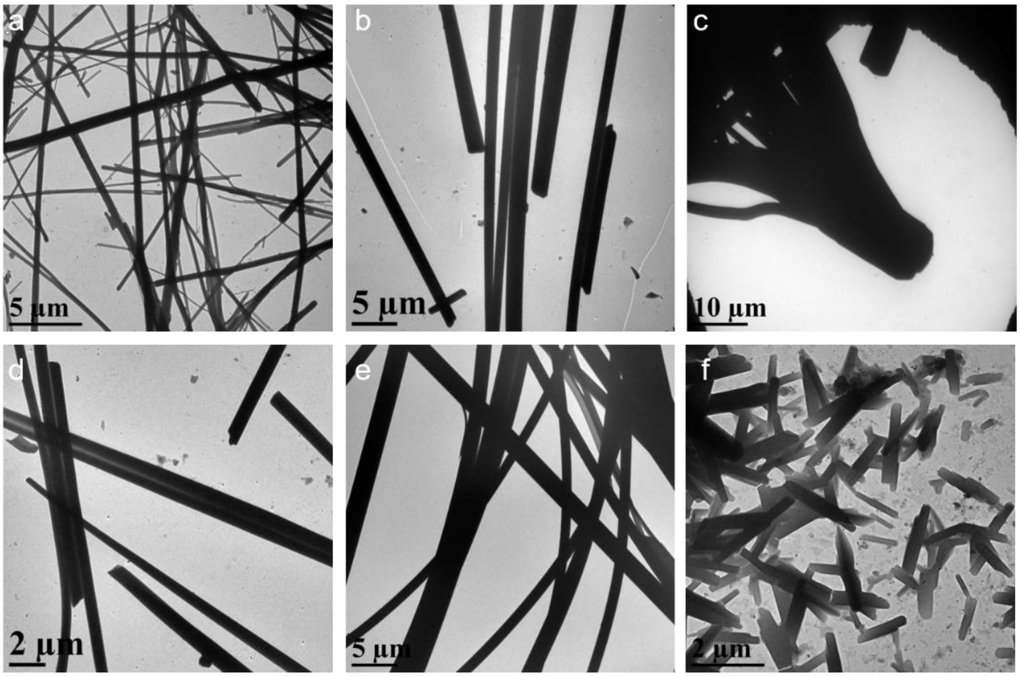

Figure S4. The TEM images of FF/NaC mixtures at NaC concentration of (**a**) 0, (**b**) 1, (**c**) 5, (**d**) 20, (**e**) 300 and (**f**) 500 mmol/L with the fixed FF concentration of 10 mmol/L.

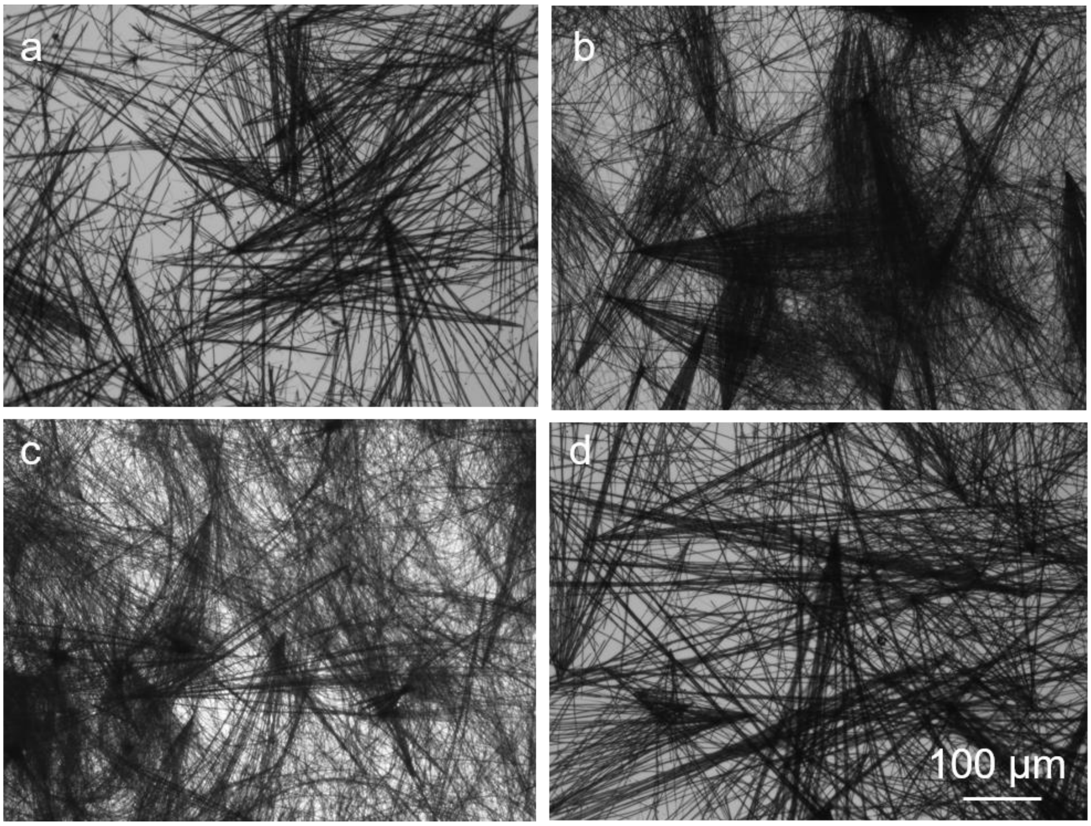

Figure S5. The images of FF/NaC mixtures at NaC concentration of (a) 5, (b) 7, (c) 10, and (d) 12 mmol/L with the fixed FF concentration of 10 mmol/L by the optical microscopy.

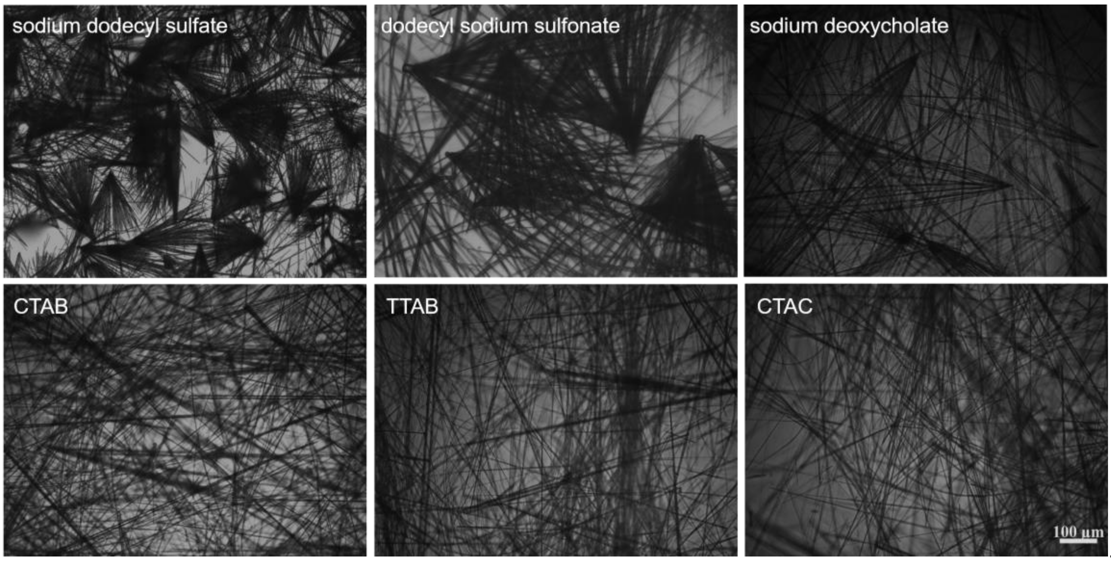

Figure S6. The images of different FF/surfactants mixtures at the concentration of surfactants near their CMC with the fixed FF concentration of 10 mmol/L by the optical microscopy. The concentration of surfactants is sodium dodecyl sulfate 9 mmol/L, dodecyl sodium sulfonate 9 mmol/L, sodium deoxycholate 4 mmol/L, CTAB 0.1 mmol/L, TTAB 0.4 mmol/L, and CTAC 16 mmol/L, respectively.
